# Elements of Physics, or Natural Philosophy, General and Medical, Explained Independently of Technical Mathematics, and Containing New Disquisitions and Practical Suggestions

**Published:** 1831

**Authors:** 


### REVIEWS

### AND BIBLIOGRAPHICAL NOTICES.

---

Art. V.—*Elements of Physics, or Natural Philosophy, General and Medical, explained independently of Technical Mathematics; and containing new disquisitions and practical suggestions.* By Neil Arnott, M. D., of the Royal College of Physicians.—First American, from the third London edition, with additions. By Isaac Hays, A. M., M. D., &c.—Philadelphia: Carey, Lea & Carey.—pp. 532.

Many reviews are written about every thing else, than the work, of which, they professedly treat. This is going too far on one side—we mean, however, to incline to the other; and if, in this notice of one of the most elegant productions of medical science, we confine ourselves, exclusively, to the work before us, our vanity leads us to hope our readers will not think that it arises from a paucity of interesting matter of our own, but from a sincere desire to call the attention of the profession to an important practical treatise.

No enlightened physician can look at the numerous classes of students which throng the halls of the various medical schools, without lamenting the great deficiency of elementary education which they exhibit. Not only are they, in general, found wanting in classical erudition, but even in the ordinary branches of school learning. How can a young man enter on the study of the most sublime of all sciences, with prospect of success, when he is absolutely ignorant of the first principles of natural philosophy, which, in the words of Lord Bacon, is *the foundation of all knowledge.*

Our medical schools too, are all defective in not appropriating a portion of the student's time to such studies. The fault lies here—Our seminaries of medical instruction are modelled after the European, in most of which, particularly the continental, the student must be a graduate in the arts before he can be suffered to matriculate as a student of medicine. We, however, take *all* in. Under these circumstances, we have long been of opinion, that a professorship of physics, or natural philosophy, ought to be attached to every regular medical school.

It is a lamentable fact that many of our respectable medical men are very imperfectly acquainted with the grand principles of natural philosophy. The application, of these great fundamental truths, to medical science, which are continually made in the various text books and other elementary works, can be but very imperfectly understood, and many theoretical and practical errors are the consequence.

The work before us is a plain treatise, calculated particularly for the medical student and practitioner. The views of the author are so fully expressed in his introduction, that we cannot do better, than to let him speak for himself:

"The author was originally led to this undertaking, with the view of supplying the desideratum in medical literature, of a treatise on *Medical Physics*; but perceiving, as he proceeded, that the preliminary investigation of *General Physics*, required to suit the work to medical readers, would require to be nearly as extensive as if the work were for general readers, he determined to make it as complete and as extensively useful as possible. He has been encouraged, during his labor, by the belief, that the growing light of science which now exhibits more clearly the natural relations of the different departments of study, as attempted to be pourtrayed in the preceding pages, might enable him to avoid some of the defects of former elementary treatises, and to add some features of novelty and improvement to his own. He thought that an elementary treatise on Natural Philosophy should be characterized—by requiring in the reader no previous information, but a knowledge of the language in which it is written, and the commonest experience of the world; by having an arrangement of the subjects, as scientific or methodical as in a strictly mathematical treatise, yet without using a single term of technical mathematics; by the general principles being illustrated in all cases, by bringing before the reader, rather interesting natural phenomena, than artificial experiments and dry abstract reasonings; by containing nothing of so little interest as to be readily forgotten; by being calculated for general readers, and not for those of one profession or class; by embracing such an account of recent improvements, as to foster the spirit which leads to further study and advancement, &c.—

In composing the *general* chapters of the work, the author, as far as his ability and leisure would permit, has been guided by these considerations. The sections on *Animal Physics* were, of course, written for medical men; and a great service will be rendered by the work, if it only awakens them to a just sense of the importance of Physics, as one of the foundations of their art. But, even for general readers, there are few parts of these sections which the author would exclude. There is nothing more admirable in nature than the structure and functions of the human body, and there are many reasons why no liberal mind should be careless of the study. The details here are not more anatomical, than the illustrations from the animal economy contained in the common treatises of *Natural Theology.*—From the attempt in this work, to compress into the smallest possible space the greatest possible sum of scientific information, few historical details have been admitted, whether relating to the distinguished men who have benefitted the world as authors or inventors, or to the history of the progress of science: such details form an interesting, but distinct branch of study. With the concluding part of the work a list will be given of the best authors who have treated on the various subjects.

The author must not conclude without observing, that no treatise on Natural Philosophy can save, to a person desiring full information on the subject, the necessity of attendance on experimental lectures or demonstrations. Things that are seen, and felt, and heard, that is, which operate on the external senses, leave on the memory much stronger, and more correct impressions, than where the conceptions are produced merely by verbal description, however vivid. And no man has ever been remarkable for his knowledge of Physics, Chemistry, or Physiology, who has not had *practical familiarity* with the objects. With reference to this familiarity, persons who take a philanthropic interest in the affairs of the world, must observe with much pleasure, the now daily increasing facilities of acquiring useful knowledge, afforded by the scientific institutions formed and forming, not only through this kingdom, but through most civilized nations.

Those of the readers of this work who know the manner of a medical man's life, will not be disappointed if they miss here the minute accuracy and polish found in the productions of more leisurely writers; but amidst the interruptions and anxieties inseparable from the author's employments, he hopes, that any defects which exist are not mixed with considerable errors. He would feel greater solicitude, as to the reception of this work, if he did not know that the subjects embraced in it are so exceedingly interesting in themselves, that when treated with ordinary clearness and precision, they never fail to please."

1*st March*, 1827.

This work, decidedly the best which has appeared for the purposes of the medical student, soon attained great popularity in Europe. It is, as yet, incomplete. The volume before us embraces three parts of the general plan—an additional volume has been published, and we anticipate the speedy fulfilment of the author's promise to bring the work to entire completion.

The first volume is said to contain "additions, by Isaac Hays, A. M., M. D., &c." Additions they certainly are; but, in our opinion, they are not only of a very useless, but of an injurious character. We allude particularly to the appendix in which the American Editor attempts to make a great display of the very thing which it is the great object of the *author* to avoid—I mean technical mathematics. The few notes that are scattered through the book, by the same hand, are feeble to the last degree. Long as the lash of criticism has been applied to our physicians, for the contemptible meanness of insinuating their names into the printed title page of an American reprint, by the booksellers suffering them to add half a dozen two-penny notes, they are still so fond of good company, that they will run all risks to get into it. Leaving, then, the *notes* and the appendix out of the question, we will turn seriously to examine the contents of the work.

At the outset, indeed in the introduction itself, Dr. Arnott expresses his views of education, particularly in relation to large portions of time, which, at all the schools and colleges, is misspent over the classics, and abstract mathematics. The doctor entertains, very nearly, the same views as our celebrated countryman, Dr. Rush. Like him, however, he does not abjure the classics, but solemnly deprecates their abuse. We are pleased to find that these views are becoming more extensive in our happy land, and that professors in colleges, and schoolmasters, are now endeavoring to direct education to its only legitimate object—the preparation of the student for the business of life.

"The notions on education prevalent in the world until recently, have been as erroneous with respect to the comparative importance of different branches of knowledge, as with respect to the order of study. Thus at many of our famed schools, and even universities, the attention has been directed almost solely either to *Languages* and *Logic*, or to *Abstract Mathematics;* the preceptors seeming to forget that these objects have no value but in their application to *Physics, Chemistry,*

*Life*, and *Mind*. The reason for bestowing much attention on the Greek and Roman languages was good some centuries ago, because then no book of value existed which was not written in one of these languages; but now the case is completely reversed, for he who learns almost any matter of science from old books, is learning error, or at the least, knowledge far short of modern erudition. As to the higher mathematics, again, while they merit great honor, as being the instrument by which many useful discoveries have been made, and the conjectures of powerful minds have been confirmed, still a very deep investigation of them is neither possible to the generality of men, nor if it were so, would it be of any utility. The mode of proceeding to which we have now alluded, is just as if a man, to whom permission were given to enter and possess a magnificent garden, on condition of his procuring a key to open the gate, and measures of all kinds to estimate the riches contained within, should waste his whole life on the road, in polishing one key, or in procuring several of different materials and workmanship, and in preparing a multiplicity of unnecessary measures. This and many similar errors, arise from men not being in general taught to carry in their minds a clear conception of the general field of human knowledge, and thence of the comparative importance of the different subdivisions,—the possession of which conception is perhaps the most valuable single acquirement which the mind can make. He whose view is bounded by the limits of one or two small departments, will probably have very false ideas even of them, but he certainly will, of other parts, and of the whole; so as to be constantly exposed to commit errors hurtful to himself or to others. His mind compared to the well-ordered mind of a properly educated man, is what the crooked and misshapen body of the mechanic, confined to certain actions and attitudes, is to the godlike form of the most perfect specimen of human nature.

The whole work, which is of a strictly didactic character, exhibits occasional flashes of genius, tending to enliven the subject, and refresh the mind. It may, indeed, be pointed out as a model of scientific writing. The style is always pure, plain, and perspicuous. There is not a sentence in the whole of it, that you must read twice to comprehend. At the same time there is a dignity manifest, which assures you that you are perusing no ordinary production. There is, but too often, an awkwardness about the style of medical writings, which betrays, not only an ignorance of the ordinary rules of composition, but even a want of that facility of expression which can be acquired by habit alone. Look, for instance, at John Hunter's writings. When Hercules was set by his mistress to spin, he did not execute his task more clumsily. There you see a mighty mind at work, laboring to bring out important ideas. But, in the "Elements of Physics," all is grace and beauty. If Dr. Arnott does not exhibit himself, with the shaggy lion's skin and knotted club of Hercules, he carries the full quiver and the silver bow of Apollo.

Our readers will not need an apology for the insertion of the following impressive and eloquent extracts:

" Physics is an important foundation of the healing art. The medical man, indeed, is the engineer pre-eminently; for it is in the animal body that true perfection and the greatest variety of mechanism are found. Where is there, to illustrate *Mechanics*, a system of levers and hinges, and moving parts, like the limbs of an animal body; where such an *hydraulic* apparatus, as in the heart and blood vessels; such a *pneumatic* apparatus, as in the breathing chest; such *acoustic* instruments as in the ear and larynx; such an optical instrument as in the eye; in a word, such mechanical variety and perfection, as in the whole of the visible anatomy! All these structures the medical man, of course, should understand, as a watchmaker knows the parts of the machine about which he is employed. The latter, unless he can discover where a pin is loose, or a wheel injured, or a particle of dust adhering, or oil wanting, &c., would ill succeed in repairing an injury; and so also of the ignorant medical man, in respect to the human body. Yet will it be believed, that there are medical men who neither understand mechanics, nor hydraulics, nor pneumatics, nor optics, nor acoustics, beyond the merest routine; and that systems of medical education are put forth at this day which do not even mention the department of *Physics!*—That such is the case, furnishes illustration of what is stated in the beginning of this essay; that the sciences and arts are progressive, and that perfect methods of education must arise gradually like all other things of human contrivance. It is within the recollection of persons now living, that political economy has been discovered to be a grand foundation of the art of government, indicating means of security against many national misfortunes common in former times; yea, against even famine and war. And the day is not distant, when the members of the medical profession generally, will understand how much the correct knowledge of animal structure, and function, and many remedies, must depend on precise acquaintance of Physics.

Besides the strictly professional matters contained in the medical sections of the present work, there are many others scattered through it which must interest the medical man; such are the subjects of *meteorology*, *climate*, *ventilation* and *warming* of dwellings, *specific gravities*, &c. But, indeed, what part of Natural Philosophy is not interesting to a medical man, since the whole is becoming every day more and more a part of a liberal education? In our cities now, and even in an ordinary dwelling house, a man is surrounded by prodigies of mechanic art; and with his proud reason, is he to use these, as careless of how they are produced, as a horse is careless of how the corn falls into his manger? A general diffusion of knowledge is changing the condition of man, and elevating the human character in all ranks of society. Our remote forefathers were generally divided into small states or societies, having few relations of amity with surrounding tribes, and their thoughts and interests were confined very much within their own little territories and rude habits. In succeeding ages, their descendants found themselves belonging to larger communities, as when the English heptarchy was united; but still remote kingdoms and quarters of the world were of no interest to them, and were often totally unknown. Now, however, every one sees himself a member of one vast civilized society, which covers the face of the earth; and no part of the earth is indifferent to him. In England, a man of small fortune may cast his looks around him, and say with truth and exultation, 'I am lodged in a house that affords me conveniences and comforts, which even a king could not command some centuries ago. Ships are crossing the seas in every direction, to bring me what is useful to me from all parts of the earth. In China, men are gathering the tea-leaf for me; in America they are planting cotton for me; in the West India islands, they are preparing my sugar and my coffee; in Italy, they are feeding silkworms for me; in Saxony, they are shearing the sheep to make me clothing; at home, powerful steam engines are spinning and weaving for me, and making cutlery for me, and pumping the mines, that minerals useful to me, may be procured. Although my patrimony was small, I have post coaches running day and night, on all the roads, to carry my correspondence; I have roads, and canals, and bridges, to bear the coal for my winter fire: nay, I have protecting fleets and armies around my happy country, to secure my enjoyments and repose. Then I have editors and printers, who daily send me an account of what is going on throughout the world, among all these people who serve me. And in a corner of my house, I have Books! the miracle of all my possessions, more wonderful than the wishing cap of the Arabian tales; for they transport me instantly, not only to all places, but to all times. By my books I can conjure up before me, to vivid existence, all the great and good men of antiquity; and for my individual satisfaction, I can make them act over again the most renowned of their exploits: the orators declaim for me: the historians recite: the poets sing: and from the equator to the pole, or from the beginning of time until now, by my books, I can be where I please.'—This picture is not overcharged, and might be much extended; such being God's goodness and providence, that each individual of the civilized millions that cover the earth, may have nearly the same enjoyments as if he were the single lord of all."

"But the enlightened Christian minister earnestly recommends the study of nature; first, because from contemplating the beauty of creation, discovered by general science, with the wisdom and benevolent design manifest in all its parts, there spring up in every undepraved mind, those feelings of delight and gratitude, which constitute the adoration of natural religion, and which form, as shown by many admirable writers on Natural Theology, a fit foundation for the sublime doctrine of immortality: and secondly, because a Revelation must be proved by the miracles which accompanied its establishment; and to enable men to distinguish between miracles and the usual course of nature, a perfect knowledge of that course, or of Natural Philosophy, is essential. All the false religions of antiquity were founded on, and upheld by pretended miracles. As regards the question of immortality, even independently of Revelation, no man who contemplates the order and beauty of the material world, and who sees the hideous deformities of the moral world—where vice so often triumphs, and modest virtue pines and dies—can for a moment believe them to be the work of the same author, unless there be an hereafter or retribution; and, feeling that eternal justice requires another state for man, he embraces with delight, the cheering promises of Christianity. There have been, however, at various times, even among Christians, sincere, but weak or ill-informed men, who decried the study of the natural sciences, as inimical to true religion—as if God's ever visible and magnificent revelation of his attributes in the structure of the universe could be at variance with any other revelation!—But such prejudices are quickly passing away. Where considerable knowledge of nature exists, debasing and gloomy superstition must cease. It is not the abject terror of a slave which is inspired by contemplating the majesty and power of our God, as displayed in his works, but a sentiment akin to the tender regard which leads a favored child to approach, with confidence, a wise and indulgent parent."

The first part of the work is devoted to the examination and explanation of the great fundamental truths of physics—particularly the nature and constitutions of the material masses which compose the universe, and the motions or phenomena which are continually going on among them.

Each section is preceded by a short summary of its contents, which is very properly called an *analysis*. This analysis, in detached portions, serves the author for a text, for each particular branch of his subject, and, as it contains, in a few words, the substance of the section, he wishes to impress it forcibly on the mind of the reader. A portion of the analysis is printed between inverted commas, at the commencement of each subject; and it is particularly requested that the student reperuse the analysis at the several interruptions, that he may have constantly before him that clear view of the general relations, among the different parts of the subject, which is essential to a perfect understanding of it.

In a review of such a treatise, it is utterly impossible that we should succeed in giving any thing like a sketch of the whole design. All we can do is to walk through the varied extent of subjects; and, here and there, stop to point out a prospect of unusual interest and beauty.

Preceded, as the animal part of the work is, by considerations of a philosophical character, it forms decidedly the best possible work on physics, to place in the hands of a medical student. No preceptor ought to suffer a student to enter on the immediate purposes of medical instruction without a sedulous and laborious perusal of this work—a work which, in our opinion, ought to receive an annual perusal from every student and every practitioner of medicine. It ought to be found on the shelves of all our country practitioners, as a book of reference. Many of our most distinguished and successful physicians of the western states are self-made men—of powerful intellect and of inventive genius. These men are mostly unacquainted with the general doctrines of physics. A work that, like this, will bring philosophy to their homes and fireplaces, is the very thing for them. It will illuminate their path, and render their assiduous labors tenfold more productive.

Nor is it to this class alone that such a work is important.—The most learned man cannot rise from the perusal of such a book, without finding himself more learned.

Dr. Arnott's is, in truth, a book suited to the character and wants of the nineteenth century. It is a plain, practical treatise, that will live and be respected, when all the visionary speculations of the theorists will have vanished, with the memory of their projectors, into "thin air." The illustrations of the grand principles of nature are drawn from the more familiar sources. We take at random his exemplifications of capillary attraction:

*Attraction* is called *capillary* when it acts between a liquid and a solid, which is tubular or porous.

When an open glass tube is partially immersed in water, the water within it stands above the level of that on the outside, and the difference of level is greater as the tube is less, because, in small tubes, the glass all round being nearer to the raised water, attracts it more powerfully.

Between two plates of glass standing near to each other, the same rising of water will occur; and if they are closer at one perpendicular edge than at the other, the surface of the suspended water will be higher there.

A piece of sponge or a lump of sugar, touching water by its lowest corner, soon becomes moistened throughout.

The wick of a lamp lifts the oil to supply the flame, from two to three inches below it.

A mass of cotton thread hanging over the edge of a glass of water will empty it, as a syphon would. A towel will empty a basin of water in the same way.

Dry wedges of wood driven into a circular groove, formed round a pillar of stone, on being moistened, will swell so as to rive off the portion from the block. Millstones are thus cut from the rock, in some quarries of Germany.

An immense weight or mass may be raised a little way, by first tightening a dry rope between it and a support, and then wetting the rope:—the moisture imbibed into the substance of the rope by capillary attraction causes it to become shorter.

At one time the small vessels of vegetables were supposed to raise the sap from the roots, by capillary attraction, but this is known now to be chiefly an action of vegetable life.

See also his account of dilatation by heat:

*Dilatation.*—A rod of iron, which, when cold, will pass through a certain opening, and will lie lengthwise between two fixed points, when heated, becomes too thick and too long to do either.

For accurate mensuration, therefore, rods or chains used as the measure, must either be at a given temperature, or due allowance must be made for the difference.

The wall of a building had begun to bulge out so as to threaten its stability. No force tried was sufficient to return it to perpendicularity, until the idea occurred of using the contracting force of iron, while cooling. The wall was connected with the opposite wall by a number of iron bars, passing through both, and having nuts screwed upon their projecting ends, which bars were then heated, one half at a time, by lamps placed under them, and while lengthened in consequence, and projecting beyond the wall, the nuts were screwed close up to the wall, so that on again cooling, and contracting, they pulled the wall back to its place.

The iron rim of a coach-wheel, when heated, goes on loosely and easily, and when afterwards cooled, it binds the wheel most tightly, giving remarkable firmness and strength.

Iron hoops on masts, and on casks, are made to bind in a similar manner.

The common thermometer, for measuring degrees of heat, is a glass bulb, filled with mercury or other fluid, and having a narrow tube rising from it, into which the fluid ascends on being expanded by heat, and so marks the degree.

A flaccid bladder of cold air, on being heated, becomes tense, and in certain cases, will burst."

We can hardly suppose Dr. Arnott ignorant of the name of the building that was thus saved by the application of this beautiful law of nature. Still less do we suspect that private or national pique would lead him to conceal that it was the Conservatory of Arts, in Paris; a building which is endeared to every lover of science, by the valuable collection of models it enshrines. It is indeed the great treasure house of the arts.

"Hic arma—hic currus."—*Virgil.*

Again:

"*Hardness*" is not proportioned, as might be expected, to the density of the different bodies, but to the polarity of the atoms in them, that is, to the force with which the atoms hold their places in some particular arrangement.

"Hardness is measured generally by the circumstance of one body being capable of scratching another.—It is worthy of notice, however, that the powder or dust of a softer body will often aid in wearing down or polishing one that is harder.

Gold, though soft, is four times heavier than the hard diamond; and mercury, which is fluid, is nearly twice as dense as the hardest steel.

Diamond is the hardest of known substances. It cuts or scratches every other body, and is generally polished by means of its own dust.

Glass-cutters use a point of diamond as a glass-knife, dividing and shaping their panes.

Common flint also cuts glass, as is seen in the common scribblings on windows.

It is remarkable, that the preparation of iron, called steel, may either be soft like pure iron, or by being heated and suddenly cooled, in the process called tempering, may become nearly as hard as diamond. The discovery of this fact is, perhaps, second in importance to few discoveries which man has made; for it has given him all the edge-tools and cutting instruments, by which he now moulds every other substance to his wishes. A savage will work for twelve months, with fire and sharp stones, to fell a great tree, and to give it the shape of a canoe; when a modern carpenter, with his tools, could accomplish the work in a day or two.

The project has lately been realized of making engravings on plates of soft steel, instead of copper, and afterwards tempering the steel to such hardness, that it may be used as a type, or die, to make its impression, not on paper, but on other plates of soft steel, or of copper; each of which is then equal in value to an original and distinct engraving; by this means the beautiful productions of art, instead of being limited to a comparatively small number of copies and persons, may be multiplied almost to infinity, becoming the chief delight of all."

As Dr. Arnott disclaims all historical detail, we could forgive his omitting to place on the brows of our countryman, Perkins, the chaplet of praise for transferring engravings from steel to copper or soft steel, were it not that, in another part of his work, he has travelled a little out of the record, to give this ingenious philosopher a rap on the knuckles for a failure. There is a decided tinge of illiberality; such, too, as we did not expect from Dr. A., in the concluding sentence:

"From misapprehension of the law of increase of force, by increase of heat, in water, explained by the table at page 329, some exceedingly false conclusions have been drawn, and acted upon at great expense—as lately by Mr. Perkins, in attempts to make engines to work with an excessively high pressure. Besides making the error now alluded to, and others, Mr. Perkins also neglected the fact, that we possess no material for cylinders and pistons strong enough to bear the contemplated pressure and friction even for a moderate time. Perhaps better proof could not be adduced of the absurdities into which even highly ingenious men may fall when ignorant of those general truths of nature on which all branches of art are founded, than the history of supposed inventions and improvements connected with the steam-engine."

We would simply state, that we happened to be in London at the very time that Perkins was making his experiments, and that we happened to know that Dr. Henry, of Manchester, and Sir Humphrey Davy both thought well of Mr. P.'s project. We also know that, as to the cylinders and pistons, the invention of the steel collars and hollow cylindric copper pistons, made by Mr. Perkins, so much surprised Bramah, that he thought his hydrostatic press would be thereby rendered many hundred times more powerful. It would be well for science, had Dr. Arnott less of the *insular* feeling.

In mentioning tenacity, our author remarks:

"Certain animal substances have great tenacity: as—the silk-worm's thread, which is our strongest conneceting or sewing material, and has such flexibility united with its strength—the ligaments and tendons of the animal body, possessing, at once, such admirable strength, elasticity, and pliancy: these, when dried, and otherwise prepared, constituted the bow-strings of our remote forefathers—the hair or wool of animals, twisted into threads, and worked into the strong and beautiful textures of the loom—strips of animal intestine, prepared and twisted, forming the cords of harp and violin, and in strength and uniformity, rivalling the steel wires of keyed instruments.

The gradual discovery of substances possessed of strong tenacity, and which man could yet easily mould and apply to his purposes, has been of great importance to his progress in the arts of life. The place of the hempen cordage of European navies is still held in China by twisted canes and strips of bamboo; and even the hempen cable of Europe, so great an improvement on former usage, is now rapidly giving way to the more complete and commodious security of the iron chain—of which the material to our remote ancestors existed only as useless stone or earth.—And what a magnificent spectacle is it, at the present day, to behold chains of tenacious iron, stretched high across a channel of the ocean, as at the Menai Strait between Anglesea and England, and supporting an admirable bridge-road of safety, along which crowded processions may pour, regardless of the deep below, or of the storm; while ships there, with sails full-spread, pursue their course, unmolesting and unmolested!"

Look at the felicitous illustrations he presents on the subject of the inertia of bodies:

"When the sails of a ship are first spread to receive the force or impulse of the wind, the vessel does not acquire her velocity at once, but slowly, as the continuing force gradually overcomes the inertia of her mass. If the sails are afterwards suddenly taken in, she does not loose her motion at once, but slowly again, as the continued resisting force of the water destroys it.

Horses must make a greater effort at first to put a carriage in motion, than to maintain the motion afterwards. And a strong effort is required to stop a moving carriage.

When a carriage hanging from springs first begins to move, the body of it appears to fall back, and a person within, seems to be suddenly thrown against the back cushion. When the carriage stops again, the body swings forward, and if the stoppage be very sudden, a careless passenger may find his head pressing through a front glass. These particulars prove the inertia, first of rest, and secondly of motion.

A man standing carelessly at the stern of a boat, when the boat begins to move, falls into the water behind; because his feet are pulled forward while the inertia of his body keeps it where it was, and therefore without its support. The stopping of a boat, again, illustrates the opposite inertia of motion, by the man's falling forward.

A bad rider on horseback may be left behind, when his horse darts off suddenly; or may be thrown off on one side by the horse starting to the other. A horse at speed, stopping suddenly, often sends his cavalier over his ears;—as was mortifyingly experienced by a coxcomb, who chose to canter along a footpath, to the annoyance of the company, and whose horse, on hearing the word *halt*, loudly addressed to it by a waggish spectator, who knew its military history, suddenly stood, and got rid of its load. The will of the beau had sinned grossly against the law of propriety, but his body very perfectly obeyed the laws of inertia and gravity, by shooting forward in its parabolic curve to the earth.

A young and not yet skilful Jehu having run his phæton against a heavy carriage on the road, foolishly and dishonestly excused his awkwardness, in a way which led to his father's prosecuting the old coachman for furious driving. The youth and his servant both deposed, that the shock of the carriage was so great, as to have thrown them over their horses' heads, and thus they lost their cause, by unwittingly proving, that the faulty velocity was their own.

A man jumping from a carriage at speed, is in great danger of falling, after his feet reach the ground; for his body has as much forward velocity, as if he had been running with the speed of the carriage; and unless he advance his feet as in running, to support his advancing body, he must as certainly be dashed to the ground, as a runner whose feet are suddenly arrested. A man racing, who receives a signal to stop, and a man jumping from a flying vehicle, must check their motion nearly in the same way.

A person wishing to leap over a ditch or chasm, makes a run first, that the motion thereby acquired may help him over. A standing leap falls much short of a running one.

An African traveller saw himself followed by a tiger, from which he could not escape by running; but perceiving that the animal was watching an opportunity to seize him by the usual spring or leap, he artfully led it to where the plain terminated in a precipice, hidden by brushwood, and he had just time to transfer his hat and cloak to a bush, and to retreat a few paces, when the tiger sprung upon the bush, and by the motal inertia of his body, was carried over the precipice, and destroyed.

From a glass of water suddenly pushed forward on a table, the water is spilt or left behind, but if the glass be already in motion, as when carried by a person walking, and if it then be suddenly stopped by coming against an impediment, the water is thrown or spilt forward.

A servant carrying a tray of glasses or china in the dark, and coming suddenly against an obstacle, hears all his freight slipping forward and crashing at his feet: and a too hurried departure with such a load causes equal destruction, on the opposite side.

The actions of beating a coat or carpet with a cane, to expel the dust; of shaking the snow from one's shoes, by kicking against the door-post; of knocking a dusty book against a table, or shutting it violently —all illustrate the same principle.

If a guinea be laid on a card, already resting on the point of the finger, a small fillip or blow to the edge of the card will cause it to dart off, but the guinea, by its inertia, will remain resting on the finger.

When we desire a person, with suspected disease of the brain, to shake his head, and tell whether and where he feels pain, we are doing nearly as if we touched the naked brain with the finger, to find the tender part; for the inertia of the brain, when the skull is moved, causes a momentary pressure between it and the skull, almost equivalent for our purpose to such a touch.

This kind of pressure is sufficient to break and destroy tender wares—as glass or eggs—in packages which are too suddenly moved or stopped.

A weight suspended by a string on shipboard, is seen vibrating up and down, as the ship pitches with the waves. It seems to fall as the ship rises, and to rise as the ship falls: but the motion is really in the ship, and the rest is in the weight. A heavy weight so supported, and connected with a pump-rod, would work the pump.

Like the weight last mentioned, the mercury of a common barometer on shipboard is seen rising and falling in the tube; and until the important improvement was lately made, of narrowing the tube in one place to prevent this, the barometer was useless at sea. The explanation is, that the tube rises and falls with the ship, from being connected with it; but the mercury, which plays freely in the tube, and is supported by the atmospheric pressure, tends, by its inertia, to remain at rest, and thus makes the motion of the ship apparent.

Like the mercury in the barometer tube on shipboard, the blood in the vessels of animals is similarly affected under similar circumstances. In a long vein below the heart, when the body falls, the blood, by its inertia and the supporting action of the vessels, does not fall so fast, and therefore really rises in the vein: and as there are valves in the veins preventing return, the circulation is thus quickened without any muscular exhaustion on the part of the individual. This helps to explain the effect of the movement of carriages, vessels at sea, swings, and of passive exercise generally, on the circulation, and leaves it less a mystery why these are often so useful in certain states of weak health.

If a cannon ball were to break to pieces in its flight, its parts would still advance with the previous velocity. Thus also, in the deadly contrivance of the Shrapnel shell, which is a case containing hundreds of musket bullets, these being set loose at the desired distance from the devoted body of men, retain the forward velocity of the shell, and spread death around like the discharge on the spot of a whole battalion of musketry.

On the awful occasion of a ship in rapid motion being suddenly arrested by a sunken rock, all things on board, men, guns, and furniture, start from their places, and dash forwards; and the inertia or motal obstinacy of the stern parts of the ship, by pressing forward, crushes the bow against the rock.

A beautiful instance is presented, of the perfection of the mechanic arts in the construction of the chronometer.

"It would be exceeding the limit marked out for this general work, to speak more particularly here of those admirable watches which have been produced within the last thirty years, under the name of *chronometers,* for the purpose of ascertaining the longitude at sea; but the author may perhaps be excused for mentioning a moment of surprise and delight which he experienced, on first seeing their singular perfection experimentally proved. After months spent in a passage from South America to Asia, his pocket chronometer and others on board, announced one morning that a certain point of land was then bearing east from the ship at a distance of fifty miles; in an hour afterwards, when a mist had cleared away, the looker-out on the mast gave the joyous call of "Land ahead!" verifying the report of the chronometers almost to a mile, after a voyage of thousands. It is allowable at such a moment, with the dangers and uncertainties of ancient navigation before the mind, to exult in contemplating what man has now achieved. Had the rate of the wonderful little instrument, in all that time, been quickened or slackened ever so slightly, its announcement would have been worse than useless,—but in the night and in the day, in storm and in calm, in heat and in cold, its steady beat went on, keeping exact account of the rolling of the earth and of the stars; and in the midst of trackless waves which retain no mark, it was always ready to tell its magic tale of the very spot of the globe over which it had arrived."

After our author has made a remarkably simple and perspicuous display of the principles which regulate the centre of gravity, he gives us a development of the actions and postures of animals, particularly of man, calculated to illustrate these principles. His remarks on seasickness are truly interesting.

"A body, we have seen, is tottering in proportion as it has great altitude and narrow base—but it is the noble prerogative of man to be able to support his towering figure with great firmness, on a very narrow base, and under constant change of attitude. The faculty is acquired slowly, because of the difficulty. A child does well who walks at the end of ten or twelve months, while the young of quadrupeds, which have a broad supporting base, are able to stand, and even to move about almost immediately.

The supporting base of a man consists of the feet and the space between them. The advantage of turning out the toes is, that without taking much from the length of the base, it adds a good deal to the breadth.

If there be considerable art in walking on two perfect feet, there must be still more in walking on two slender wooden legs with rounded extremities:—which we often see done, nevertheless, by mutilated soldiers and sailors.

All the ladies of the empire of China have to acquire nearly the same talent as these victims of war; for barbarous custom has crippled them, for confining their feet for life, in the shoes which fitted them in infancy.

But surpassing in difficulty any of these instances, is the practice of walking on stilts, which is general among the inhabitants of the sandy plains called *Les Landes*, in the south-west of France. These plains afford tolerable pasturage for sheep; but during one portion of the year they are half covered with water, and during the remainder they are still very unfit walking ground, by reason of their deep loose sand and thick furze. The natives combat the inconveniences of all the seasons, by doubling the length of their natural legs, through the addition to them of the stilts mentioned, which they call *des echasses*. These are wooden poles, put on and off as regularly as the other parts of their dress. The people, when raised upon them, appear to strangers a new and extraordinary race of long-legged beings, marching readily over the loose sand, or through the water, with steps of eight or ten feet in length, and with speed equalling that of a trotting horse; their moderate journeys being of thirty or forty miles in a day. The shepherds, while watching their flocks, post themselves in convenient stations, with a long staff to support them behind, and having a rough sheepskin cloak and cap to cover them above, like a thatched roof, they appear like little watch towers, or singular lofty tripods, scattered over the face of the country.

Still beyond the art of walking on stilts is that which some persons attain of walking and dancing on a single rope or wire; or even of keeping the centre of gravity above the base, while standing on the moveable support of a galloping horse. The rope dancer usually carries a long pole in his hand to balance him; it is loaded at each end, and when he inclines, he throws it a little towards the side required, that the reaction may restore his body to perpendicularity.

Much art of the same sort is shown in the attitudes and evolutions of the skater; in the amusements of supporting a stick upright on the end of the finger; and in many other feats of a like kind.

*Attitudes* generally depend on the necessity of keeping the centre of gravity of the body over the base under variety of circumstance.—The following are examples:—the straight or upright port of a man who carries a load on his head;—the leaning forward of one who carries it on his back;—the hanging backwards of one who bears it between his arms;—the leaning to one side of him who is carrying a weight on the other side;—the habitual carriage of very fat people, whose head and shoulders are thrown back, giving a certain air of self-satisfaction,—an air which belongs also to a state of pregnancy, and even to that of the dropsical patient, although producing in it so sad an incongruity.

When a man walks or runs, he inclines forward, that the centre of gravity may overhang the base; and he must then be constantly advancing his feet to prevent his falling. He makes his body incline just enough to produce the velocity which he desires.

A man, in pulling horizontally at a load, is merely causing his body to overhang its base, so that its tendency to fall may become a force or power applicable to the work.

When a man rises from a chair, he is seen first bending the body forward, so as to bring the centre of gravity over the feet or base, and then he lifts the body up. If he lift too soon, that is, before the body be sufficiently advanced, he falls back again.

A man standing with his heels close to a perpendicular wall, cannot bend forward sufficiently to pick up any object that lies on the ground near him, without himself falling forward; because the wall prevents him from throwing part of his body backward, to counterbalance the head and arms that must project forward. A man little versed in such matters, agreed to give ten guineas for permission to try to possess himself of a purse of twenty, thus laid before him; he of course lost his money.

When a man walks at a moderate rate, his centre of gravity comes alternately over the right and over the left foot. This is the reason why the body advances in a waving line, and why persons walking arm in arm shake each other, unless they make the movements of their feet to correspond, as soldiers do in marching.

*Seasickness* is a subject closely related to the present. Man requiring, as now explained, so strictly to maintain his perpendicularity, that is, to keep the centre of gravity always over the support of his body, ascertains the required position in various ways, but chiefly by the perpendicularity or known position of things about him. Vertigo, and sickness, are the consequences of depriving him of his standards of comparison, or of disturbing them.

Hence on shipboard, where the lines of the masts, windows, furniture, &c., are constantly changing, sickness, vertigo, and other affections of the same class, are common to persons unaccustomed to ships. Many experience similar effects in carriages, and in swings; or on looking from a lofty precipice, where known objects being distant, and viewed under a new aspect, are not so readily recognized; also in walking on a wall or roof; in looking directly up to a roof, or to the stars in the zenith, because then all standards disappear: on entering a round room, where there are no perpendicular lines of light and shade, as when the walls and roof are covered with a paper which has no regular arrangement of spot: on turning round, as in waltzing, or if placed on a wheel; because the eye is not then allowed to rest long enough on any standard, &c.

All people in the dark, and therefore blind people always, use standards belonging to the sense of touch; and it is because, on board of ship, the standards both of sight and of touch are lost, that the effect is so very remarkable.

But seasickness also partly depends on the irregular pressure of the bowels among themselves and against the containing parts, when their inertia or downward pressure varies with the rising and falling of the ship.

From the nature of seasickness, as discovered in these facts, it is seen why persons unaccustomed to the motion of a ship, often find relief by keeping their eyes directed to the fixed shore, where it is visible; or by lying down on their backs and shutting their eyes; or by taking such a dose of exhilarating drink as shall diminish their sensibility to all objects of external sense."

"*Beauty of attitude* and *grace of carriage* in the human individual, are in great part referable to this principle.

The postures of opera dancers might pass as intentional illustrations of the number of ways in which the centre of gravity may be kept above a narrow base, by counteracting one disturbing motion or extension of a limb by some opposite and corresponding motion. The common statue of the god Mercury on tiptoe is a permanent familiar illustration of a beautifully balanced attitude.

Grace of carriage includes not only a perfect freedom of motion, but also a firmness of step, or steady bearing of the centre of gravity over the base. It is usually possessed by those who live in the country, and according to nature, as it is called, taking much and varied exercise. What a contrast is there between the gait of the active mountaineer, rejoicing in the consciousness of perfect nature, and that of the mechanic or shopkeeper, whose confinement to the cell of his trade, soon produces in his body a shape and air that correspond to it: —and in the softer sex what a contrast is there, between the fair one who recalls to us the fabled Diana of old, and that other, who having scarcely trodden but on smooth pavements or carpets, under any new circumstances, carries her person as if it were a load altogether new and foreign to her."

Having given the laws of mechanics, Dr. Arnott proceeds to apply them to the mechanism of the human skeleton. Such applications are very frequently to be met with, in works on anatomy and physiology; but, not in the style in which they are here given. There is a raciness in Dr. A.'s matter and manner, that keeps the appetite whetted. For even on the most trite subjects, one feels as little inclined to close his book, as does a boarding-school miss to fall asleep over a new novel. Our limits will not permit us to give samples of this part. In our January number, we presented a review of Mr. Duffin's little work on deformity of the spine. The following extract on the same subject, from our author, will form a supplement of a very useful character. Premising a series of observations on the elasticity, flexibility, and strength of the spinal column, he presents the following considerations:

"To the well-being of the higher classes of animals, exercise of their various parts is as necessary as their nourishment, and if it be withheld by any cause during the period of growth, the body is often permanently crippled, or at least, never acquires its due form and proportions. The overflow of life and energy which nature has given to young creatures, to prompt them to the required exertion, is seen in the ever-changing occupation of a child, the quick succession of its ideas, its jumping and skipping, and using all the modes of round-about action that will expend muscular energy, instead of seeking, as in after life, to accomplish its desired ends in the shortest ways:—and among the inferior animals, the same truth is illustrated by the play of kittens, puppies, lambs, &c. But, strongly as nature has thus expressed herself upon the important subject of exercise among the young, tyrant fashion, with a usual perversion of common sense, has of late times, in England, formed a school of discipline, for young women of the higher classes, which wars directly with nature's dictate. The consequences have been such, that a stranger arriving here from China, might almost suppose it the design to make crooked and weak spines by our school discipline, as it is the design in China to make little feet by the iron shoe. The result is the more striking, because the brothers of the female victims, and who of course have similar constitutions, are robust, healthy, and well formed. A *peasant girl*, when her spirits are buoyant, is allowed to obey her natural feeling, and at proper times may dance, and skip, and run, until healthy exhaustion asks that repose, which is equally allowed; and thus she grows up strong and straight: but the *young lady* is receiving constant admonition to curb all propensity to such vulgar activity, and often, just in proportion as she subdues nature, she receives the praise of being *well bred*. The multifarious studies of the young lady come powerfully in aid of the admonition, by fixing her for many hours every day to sedentary employment. The consequences soon follow, of weakness in the body generally from the want of the natural quantity and variety of exercise, but of the back particularly, from the manner in which the sitting is usually performed. While it would be accounted great cruelty to make a delicate young creature stand all day, because the legs would tire, this very cruelty is in almost constant operation against the back, as if backs could not tire as well as legs. A girl is allowed to sit down because she has been long standing, but great care is taken that the muscles of the back, which still remain in action as she sits, shall not be at all relieved; for, from the idea that it is ungraceful to loll, she is either put upon a stool which has no back at all, or upon a very narrow chair with a perpendicular back. Now neither of these relieves her spine, yet the stool is less hurtful than the chair, by allowing it to bend in different ways, so as to rest the different sets of muscles alternately, while the chair keeps it constantly upright, and nearly unmoved. The excessive fatigue soon causes the spine to give way in some part, and to bend, and the curvature becomes permanent. When a bend has taken place in one situation, there immediately follows an opposite bend above or below, to keep the centre of gravity of the body always directly over the base. Thus the curve becomes double, like an italic *f*, and the distortion is complete. In bending, the spine is sometimes also partially rotated, so as to show from behind that waving profile which should be seen only from the side.

In the school discipline now spoken of, when the inclination of the back has once begun, it is very soon increased by the means used to cure it. Strong, stiff stays, are put on, to support the back as it is said, but which in reality, by superseding the action of the muscles intended by nature as the supports, cause these to loose their strength, so that when the stays are withdrawn, they are unable to support the body. Longer sittings in the narrow upright chair are then recommended, and sometimes the back is forcibly stretched by pulleys, or the patient is kept all day and night lying on an inclined board, losing her health, &c. &c. The only things guarded against are the allowing to the child proper exercise and air, and the letting her rest when she is not taking exercise. With many persons the prejudice had at least grown up, that strong stays should be put on at a very early age, to prevent the first approach of the mischief, and that a child should always be made to sit on the straight-backed chair or to lie on the hard plane: and it is probable, that if these cures and preventives had been adopted as universally and strictly as many deemed them necessary, we should now scarcely have in England a young lady with a back strong enough properly to perform its offices. It would be disgusting to see an attempt made to improve the strength and shape of a young race horse or greyhound, by binding tight splints or stays round its beautiful young body, and then tying it up in a stall; but this is the kind of absurdity and cruelty which has been so commonly practiced in this country, towards what may well be called the most faultless of created beings."

The mechanism of the heel deserves our notice. The facts here enumerated deserve the attention of parents, as bearing immediately on that much neglected part of parental duty—the physical education of children. How many a beautiful foot is spoiled, how many an awkward gait contracted, by the palpable neglect of parents! The good doctor seems to speak with a feeling of enthusiasm, of the Parisian ladies' legs, slightly tinged, however, with sectional dislike of their city.

"*The heel*, by projecting so far backwards, is a lever for the strong muscles to act by, which form the calf of the leg, and terminate in the tendo achillis. These muscles, by drawing at it, lift the body, in the actions of standing on the toes, walking, dancing, &c. In the foot of the negro, the heel is so long as to be ugly in European estimation; and its great length rendering the effort of smaller muscles sufficient for the various purposes, the calf of the leg in the negro is smaller in proportion than in other races of men.

In a graceful human step, the heel is always raised before the foot is lifted from the ground, as if the foot were part of a wheel rolling forward; and the weight of the body supported by the muscles of the calf of the leg, as just described, rests for the time on the forepart of the foot and toes. There is then a bending of the foot in a certain degree. But where strong wooden shoes are used, or any shoe so stiff that it will not yield and allow this bending of the foot, the heel is not raised at all until the whole foot rises with it, so that the muscles of the calf are scarcely used, and in consequence soon dwindle in size, and almost disappear. Many of the English farm servants wear heavy stiff shoes; and in London it is a striking thing to see the drivers of country wagons, with fine robust persons in the upper part, but with legs which are fleshless spindles, producing a gait most awkward and unmanly. The brothers of these men, and who are otherwise employed, are not so misshapen. What a pity that, for the sake of a trifling saving, fair nature should be thus deformed. An example of an opposite kind is seen in Paris. There, as the streets have no side pavements, and the ladies have consequently to walk almost constantly on tiptoe, the great actions of the muscles of the calf has given a conformation of the leg and foot, to match which the Parisian belles proudly challenge all the world, not aware probably, that it is a defect in their city to which the peculiarity of their form is in part owing."

A single extract on the subject of dislocations:

"In the treatment of dislocations and fractures of the frame-work of the human body, the surgeon cannot avoid displaying strikingly, either his skill in his profession, or his ignorance. With what ease does the displaced arm or thigh-bone return, under the guidance of the skilful hand! and to what horrible and unavailing torture is the victim subjected, when in such a case ignorance dares the attempt!—It is positive pain to a vivid imagination to look into the records of ancient surgery, and to be made present, as it were, to the stretching of patients on the rack with pullies and powerful engines, to do what better information could so easily have accomplished without violence. And would that the records of modern times contained fewer instances of individuals crippled for life by such practice. Nothing can now ensure impunity and a quiet conscience to a practitioner in this branch, if he wants a familiarity with the laws of mechanical philosophy, and a perfect knowledge of anatomy.

With our present information on these subjects, we are surprised at the details of the practices and errors promulgated in former times, owing to ignorance of mechanics, even by authors of the highest credit. It would hardly be believed that Mr. Pott, one of the glories of English surgery, held, that in reducing a dislocation of the shoulder or hip, it was useless to pull by the hand or foot, because the intervening joints prevented the strain from reaching the part desired.

Some surgeons, possessing a certain degree of knowledge in mechanics, but only that degree which is dangerous, having heard that the lever was a powerful engine, have tried to replace bones solely by leverage, as it was called. Thus, a man's dislocated arm has been placed over the back of a chair as a fulcrum, or over the top of a door, and while the weight of the suffering body was hanging to it on one side as the resistance, force has been applied to the other side, enough sometimes to break the bone, or to tear away the ligaments and soft parts about the joint.

Other surgeons, after learning in the same way the effects of the pulley, having wished to do all by irresistible extension, and instead of borrowing the moderate assistance which might be useful, have torn muscles and ligaments from their attachments."

*(To be Continued.)*

